# *Legionella pneumophila* strain associated with the first evidence of person-to-person transmission of Legionnaires’ disease: a unique mosaic genetic backbone

**DOI:** 10.1038/srep26261

**Published:** 2016-05-19

**Authors:** Vítor Borges, Alexandra Nunes, Daniel A. Sampaio, Luís Vieira, Jorge Machado, Maria J. Simões, Paulo Gonçalves, João P. Gomes

**Affiliations:** 1 Bioinformatics Unit and Research Unit, National Institute of Health, Lisbon, Portugal; 2 Innovation and Technology Unit, National Institute of Health, Lisbon, Portugal; 3 Coordination of the Department of Infectious Diseases, National Institute of Health, Lisbon, Portugal; 4 National Reference Laboratory for Legionella, National Institute of Health, Lisbon, Portugal

## Abstract

A first strong evidence of person-to-person transmission of Legionnaires’ Disease (LD) was recently reported. Here, we characterize the genetic backbone of this case-related *Legionella pneumophila* strain (“PtVFX/2014”), which also caused a large outbreak of LD. PtVFX/2014 is phylogenetically divergent from the most worldwide studied outbreak-associated *L. pneumophila* subspecies *pneumophila* serogroup 1 strains. In fact, this strain is also from serogroup 1, but belongs to the *L. pneumophila* subspecies *fraseri*. Its genomic mosaic backbone reveals eight horizontally transferred regions encompassing genes, for instance, involved in lipopolysaccharide biosynthesis or encoding virulence-associated Dot/Icm type IVB secretion system (T4BSS) substrates. PtVFX/2014 also inherited a rare ~65 kb pathogenicity island carrying virulence factors and detoxifying enzymes believed to contribute to the emergence of best-fitted strains in water reservoirs and in human macrophages, as well as a inter-species transferred (from *L. oakridgensis*) ~37.5 kb genomic island (harboring a *lvh/lvr* T4ASS cluster) that had never been found intact within *L. pneumophila* species. PtVFX/2014 encodes another *lvh*/*lvr* cluster near to CRISPR-associated genes, which may boost *L. pneumophila* transition from an environmental bacterium to a human pathogen. Overall, this unique genomic make-up may impact PtVFX/2014 ability to adapt to diverse environments, and, ultimately, to be transmitted and cause human disease.

Legionnaires’ disease (LD) is a severe pneumonia with a case fatality rate of 8–12%, reaching higher values in elderly, smokers, and people with preexisting medical conditions, such as chronic cardiovascular or respiratory disease and diabetes^1^,^2^. LD is caused by *Legionella* spp., with *L. pneumophila* serogroup 1 accounting for most of the diagnosed cases^3^,^4^,^5^. *L. pneumophila* is a facultative intracellular Gram-negative aerobic bacterium that is widespread in nature, where water is its major reservoir, growing at the temperature range from 25 °C to 42 °C^6^. Transmission usually occurs by inhalation of aerosols or aspiration of water containing these bacteria^6^,^7^. Nevertheless, contrarily to what has been historically postulated, we have recently reported that person-to-person transmission of LD may occur^8^. LD has an incubation period typically ranging from 2–10 days, although longer and shorter periods have been described^2^. The vast majority of LD cases is sporadic, but large outbreaks may also occur^9^,^10^,^11^,^12^,^13^. The first reported outbreak dates back to 1976 and affected the members of an American Legion convention in Philadelphia, from which resulted the current disease designation^14^. The most common sources of outbreaks are contaminated cooling towers and hot water plumbing infrastructures, but decorative fountains, whirlpools or home showers^15^ have also been involved^2^. While the standardized sequence-based typing (SBT) has been useful for outbreak source identification and to study the molecular epidemiology of LD^16^,^17^, whole-genome sequencing (WGS) is emerging as a more informative technology^18^,^19^,^20^,^21^ since it enables an in-depth investigation of outbreak-related strains and a higher resolution power for source attribution. It is also imperative to use WGS to deeply genetically characterize the strains associated with either endemic cases or large outbreaks. In fact, the conjugation of these two research areas (molecular epidemiology and comparative genomics) may considerably enhance our knowledge on LD and, thus, improve public health interventions.

One of the largest outbreaks of LD worldwide occurred in 2014 in Vila Franca de Xira (VFX), Portugal, yielding more than 400 cases and 14 deaths^22^. Epidemiological, environmental, microbiological and sequence-based molecular analyses traced industrial wet cooling systems as the potential source of infection and revealed that the outbreak-related strain (here designated PtVFX/2014) was a *L. pneumophila* serogroup 1 displaying the novel sequence type (ST) 1905^13^. Remarkably, it was possible to implicate this *L. pneumophila* ST1905 strain into the strongest evidence to date of person-to-person transmission of LD^8^. Considering the huge genetic diversity within the *L. pneumophila* species^11^,^23^,^24^,^25^,^26^,^27^, marked by the presence of key virulence traits (like the well-described Dot/Icm type IVB secretion system – T4BSS) encoded in both core- and accessory-genomes^28^,^29^, we aimed to deeply characterize the genetic backbone of this novel person-to-person transmission- and outbreak-related strain by integrating it in the frame of the species phylogeny and diversity.

## Results and Discussion

### WGS-based outbreak investigation

In 2014, Portugal hosted the second largest outbreak worldwide of LD so far, which was caused by a novel ST1905 *L. pneumophila* serogroup 1 strain (PtVFX/2014) likely originated from local industrial cooling towers^13^. WGS was applied to strengthen the investigation of this LD outbreak as it provides a higher level of resolution than the traditional SBT scheme^18^,^19^,^20^,^21^, which is based on the sequence of seven loci (involving only ~2500 bp out of the ~3.4 Mbp of the *L. pneumophila* genome)^16^,^17^. The draft genome sequences of the outbreak-related strains were ~3.47 Mb in length. We found no nucleotide differences after performing pairwise comparisons between the environmental isolate and each clinical sample (using near-complete genome sequences; >99.7% of each draft genome), which suggested an industrial cooling tower as the potential source of the LD outbreak. The scenario of genomic identity among same-outbreak isolates observed here and in several other LD outbreaks is expected^9^,^10^,^21^, but contrasts with the recent findings described by Sánchez-Buso and colleagues^12^, who remarkably found non-clonal relationships among isolates epidemiologically linked to the same outbreak. In the LD outbreak occurred in Portugal, the observed clonality likely reflects the existence of a single physical environmental source with a *L. pneumophila* population markedly represented by the causative infection clone.

Of note, confirmation of the SBT-based ST1905 allelic profile highlighted a bias associated with the *in silico* extraction of the allele sequence from WGS data, as previously noticed by other authors^18^. In fact, the PtVFX/2014 strain displays two non-matching *mompS* copies (assigned with distinct allele numbers, 15 *versus* 7), which would hamper a proper ST attribution if the allelic profile was exclusively determined *in silico*.

Taken together, WGS enabled an in-depth investigation of the recent LD outbreak that occurred in Portugal, as it allowed the identification, with a high level of certainty, of an industrial cooling tower as the source of the epidemic.

### SBT-based integration of PtVFX/2014 within the worldwide *L. pneumophila* genetic diversity

**Figure 1:**
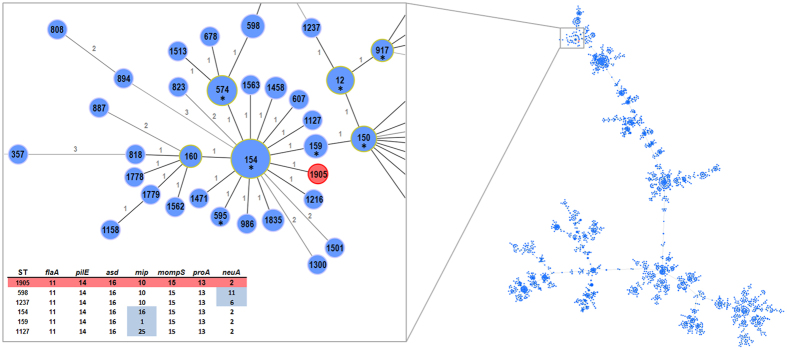
SBT-based representation of the worldwide genetic diversity among *L. pneumophila* isolates. Relationships between allelic profiles of 10085 isolates (from the EWGLI SBT database) are shown in a goeBURST full minimum spanning tree (MST) constructed using PHYLOViZ software. The MST connects the ST profiles in a way that the summed distance of all links of the tree is the minimum. STs are indicated by numbers within circles, where connecting lines are labeled with the number of allelic differences between STs. The cluster including the PtVFX/2014 ST1905 strain (in red) is zoomed, where STs found in more than one country are labeled with an asterisk. The matrix describes the ST profiles differing by a single allele from the ST1905.

In order to integrate the novel ST1905 in the worldwide epidemiological picture of the *L. pneumophila*, the allelic profile of PtVFX/2014 was compared with all STs available at the European Working Group for *Legionella* Infections (EWGLI) SBT database^16^,^17^ (Fig. 1). ST1905 was found to differ just by one allele from ST154, ST159 and ST1127 (in *mip* locus), as well as from ST598 and ST1237 (in *neuA* locus). Nevertheless, these allelic profiles differ by seven to eight SNPs to ST1905, with exception of ST159 which displays only one nucleotide difference, being thus the closest ST to ST1905 (data not shown). No linkage can be made between the geographical source and the cluster enrolling these most close related STs, as isolates from these STs were already detected in worldwide spread countries (e.g., USA, Canada, UK, Italy, France, China, Japan, among others). For instance, ST159 has been described both in Europe and Asia.

### Integration of the genetic backbone of the PtVFX/2014 strain in the frame of the species phylogeny and diversity

To get insight on the genetic backbone of the *PtVFX/2014* strain and to infer its position within the phylogeny of *L. pneumophila* species, we constructed a core-genome alignment (encompassing both coding and non-coding regions) that comprises ~64% of the genome sequence of each of the 11 outbreak-associated clones and of the 24 selected publicly available strains. This comparative analysis enrolls isolates from different *L. pneumophila* subspecies (*pneumophila*, *fraseri* and *pascullei*) that were specifically selected in order to handle as much genetic diversity as possible. This contrasts with most of the genome-based comparative studies performed so far, which have been mainly focused on *L. pneumophila* subsp. *pneumophila* (mainly through the analysis of core genes)^24^,^25^,^26^,^30^,^31^.

**Figure 2:**
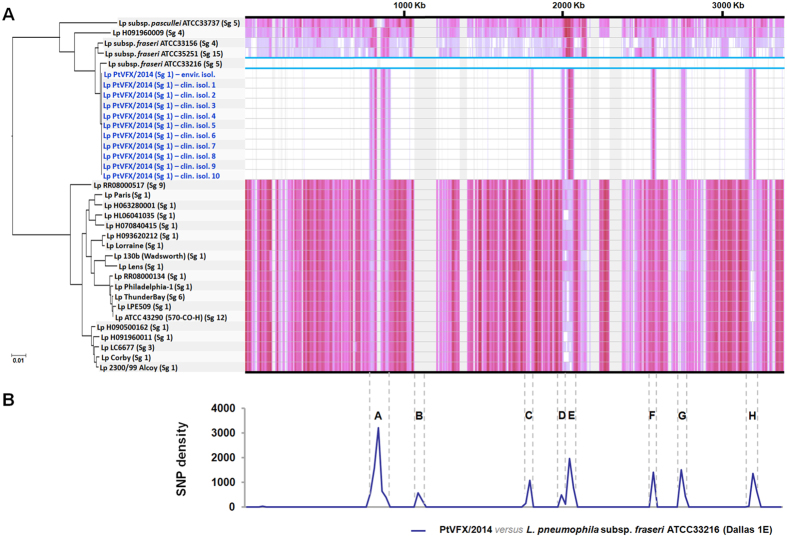
Phylogenomic analysis of PtVFX/2014 strain within the *L. pneumophila* species. (**A**) A phylogenetic reconstruction enrolling genome sequences from 24 *L. pneumophila* strains and from 11 outbreak-associated PtVFX/2014 clones (ten clinical isolates plus one environmental isolate; in dark blue) is shown paired with the corresponding SNP density plot across the length of the genome. Serogroup (Sg) classification is also shown (see Supplementary Table S1 for details). The tree is drawn to scale, with branch lengths reflecting number of base substitutions *per* site. SNP density in the core-genome (~64% of the genome) is depicted proportionally to the color intensity and reflects SNPs against the genome of the *L. pneumophila* subsp. *fraseri* ATCC33216 strain (surrounded with a light blue box). Areas in grey refer to regions in the accessory genome. Genome orientation is according to that of the *L. pneumophila* Philadelphia-1 strain (GenBank accession number AE017354). (**B**) Detailed pairwise SNP density plot between PtVFX/2014 and the most close related *L. pneumophila* subsp. fraseri ATCC33216 strain showing putative HGT-inherited regions (labeled from A to H; see details in Table 1). Calculations of SNP density were performed across the genome alignment over a sliding window (window size = 25000 bp; step size = 25000 bp). Vertical dashed lines delimiting each region make the correspondence with the SNP density plot of panel (**A**).

Intriguingly, the serogroup 1 PtVFX/2014 clones were found to be phylogenetically close related with strains from *L. pneumophila* subsp. *fraseri* (Fig. 2A), and were clearly distinct from the most worldwide studied *L. pneumophila* serogroup 1 strains (Philadelphia-1, Paris, Lens, Corby, and 2300/99 Alcoy). For instance, we detected 224376 variant sites (within a ~87% core-genome) when comparing the PtVFX/2014 strain with the well-studied serogroup 1 *L. pneumophila* Philadelphia-1 strain, whereas the pairwise genome comparison against the most close related *L. pneumophila* subsp. *fraseri* ATCC33216 (or Dallas 1E) strain revealed just up to 17000 nucleotide differences within a ~98% core-genome. SNP density analysis throughout the genome revealed a non-random distribution, as those SNPs are clustered into eight distinct chromosome regions (Fig. 2A,B), which strongly suggests their inheritance by horizontal gene transfer (HGT). Overall, these HGT-inherited regions involve around 250 genes and represent ~8% of the genome length (Table 1). One of the clearest examples of a crossover region (affecting the gene *lpg1817*), where PtVFX/2014 genome abruptly exhibits a high concentration of homoplasic mutations, is shown in Supplementary Figure S1. This is not surprising as it is well-known that recombination events and HGT are major driving forces in shaping the highly dynamic *L. pneumophila* genomes^12^,^23^,^24^,^25^, with each strain possessing its own unique genomic mosaic as an outcome of its environmental context and its specific adaptive needs to infect different hosts or to persist in the environment^32^,^33^. For instance, recombination was found to be responsible for introducing genetic variation even in a *L. pneumophila* population markedly associated with recurrent outbreaks, with implications in public health interventions for outbreak investigation and source attribution^12^.

**Figure 3:**
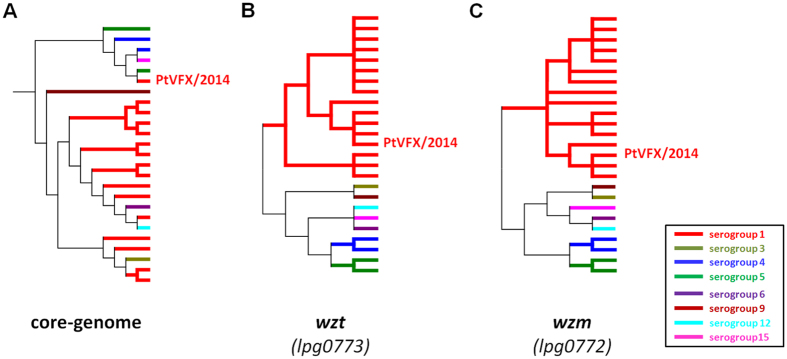
Genetic basis underlying the serogroup classification of PtVFX/2014. Schematic non-scaled condensed trees showing the genetic relatedness among 25 *L. pneumophila* strains (Supplementary Table S1) based on the core-genome alignment from Fig. 2A (panel **A**), as well as on the individual alignments of two genes that have been applied for genetic serogroup classification: *lpg0773/wzt* (panel **B**) and *lpg0772/wzm* (panel **C**). For simplification purposes, only one clone of the serogroup 1 PtVFX/2014 strain is presented in all trees. The core-genome tree topology matches the one of Fig. 2A, while the *lpg0773/wzt* and *lpg0772/wzm* trees topologies were inferred in MEGA5 using the Neighbor-Joining method, with Kimura 2-parameter model for estimating evolutionary distances (Bootstrapping = 1000 replicates). Branches are colored according to the serogroup classification.

Among the *loci* imported from the detected HGT events in the PtVFX/2014 strain (Table 1), we highlight genes coding for proteins: *i*) from the lipopolysaccharide (LPS) biosynthesis cluster; *ii*) potentially associated with resistance to antibiotics and toxic compounds, or with iron acquisition; *iii*) that likely impact the bacterium ability to infect macrophages; and, *iv*) that are potentially essential for viability and cell to cell migration of *L. pneumophila*. Phylogenetic analysis of two genes (*lpg0773*/*wzm* and *lpg0772*/*wzt*) from the LPS cluster that have been used for genetic serogroup classification^34^,^35^ strongly sustains that the HGT inheritance of the LPS cluster is responsible for the serogroup 1 profile of PtVFX/2014 (Fig. 3). This is especially relevant since *L. pneumophila* serogroup 1 isolates are predominantly reported in LD cases worldwide^3^,^5^, although PtVFX/2014 is evolutionarily divergent from the *L. pneumophila* subsp. *pneumophila* serogroup 1 strains that have caused most of large outbreaks of LD described so far^9^,^10^,^24^,^26^,^36^. On the other hand, it corroborates that the LPS cluster determining serogroup 1 can be present in highly diverse genomic backbones, and that itself likely constitutes a major determinant of human disease^35^. It is also worth noting that this HGT-derived region also involves the *inaAB* locus, which was shown to be required for assimilation, intracellular infection and virulence^37^, as well as a gene (*lpg0742*/*ldsA*) coding for a protein that may affect virulence by interfering with the assembly and/or the activation of the Dot/Icm system^38^.

Also important was the exchange of multiple genes encoding substrates of the Dot/Icm T4BSS (Table 1). It is known that critical virulence traits of bacterial pathogens rely on a wide variety of secretion systems that translocate effector proteins into the host cell in order to promote infection and survival^43^,^44^, where the Dot/Icm T4BSS is the major essential virulence factor in *L. pneumophila*^28^,^44^,^45^. In this regard, those recombination events involving Dot/Icm T4BSS effectors may impact the PtVFX/2014 ability to infect, to be transmitted, and to cause disease. For instance, we highlight two exchanged Dot/Icm effectors (Lpg0733/RavH and Lpg2461) that were previously implicated in the modulation of eukaryotic vesicular trafficking^46^, and the protective B-cell antigen Lpg2271^47^,^48^ (Table 1). Of note, multiple homoplasic mutations shared with the most worldwide studied outbreak-associated *L. pneumophila* serogroup 1 strains were detected in the genes encoding the Dot/Icm substrates Lpg2271 and Lpg1639. The mutational scenario observed for *lpg1639* (Supplementary Fig. S2) was even more marked, since PtVFX/2014 is clearly divergent from the close-related strains from *L. pneumophila* subsp. *fraseri* or *L. pneumophila* subsp. *pascullei* in this gene. Given their relevance, a detailed description of the repertoire of known Dot/Icm effectors carried by the PtVFX/2014 strain is presented below. Taken together, these results point that the outbreak-related PtVFX/2014 strain acquired by HGT multiple genetic features that may be associated with specific virulence or transmission traits.

### Other virulence-associated traits of the *L. pneumophila* PtVFX/2014 strain

**Figure 4:**
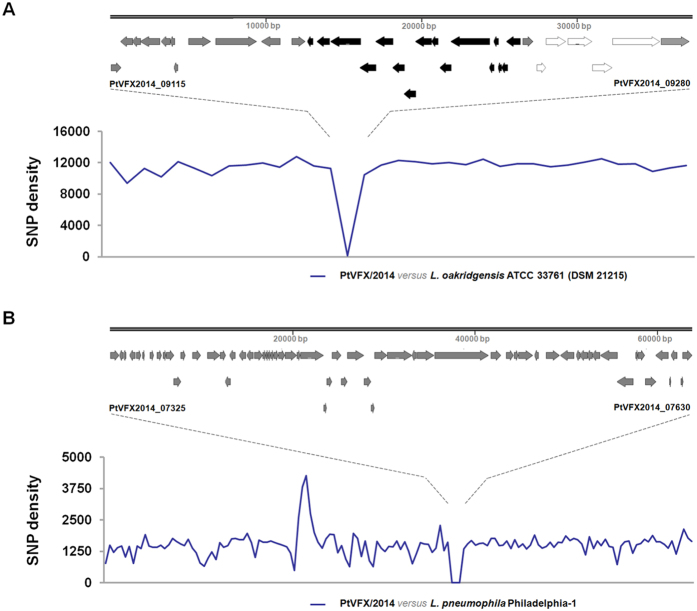
Relevant putative pathogenicity islands inherited by PtVFX/2014. (**A**) The graph shows the SNP density across a core-genome alignment (encompassing about 39–49% of each whole-genome sequence) between PtVFX/2014 and the *L. oakridgensis* ATCC 33761 (or DSM 21215) strain over a sliding window (window size = 38000 bp; step size = 38000 bp). Both gene content and organization of the ~37.5 kb genomic island is highlighted above the graph, where the *lvh/lvr* cluster is shown in black arrows, while the gene cluster homolog to a type I-like restriction-modification system is displayed in white arrows. (**B**) The graph shows the SNP density across a core-genome alignment (encompassing about 86–88% of each whole-genome sequence) between PtVFX/2014 and the *L. pneumophila* Philadelphia-1 strain over a slinding window (window size = 20000 bp; step size = 20000 bp). Gene organization of the ~65 kb genomic island is highlighted above the graph. For both graphs, core-genome alignments were extracted (using MAUVE software) by keeping and concatenating regions where genomes aligned over at least 500 bp, x-axis do not reflect any genome orientation. Genes delimiting each genomic island are labeled in each panel.

It is well-known that the *L. pneumophila* genome is highly plastic, with an accessory genome marked by several pathogenicity islands, plasmid-like elements, phage-related genes and other virulence-associated loci potentially related to its ability to adapt and survive in diverse environments and to cause human disease^12^,^20^,^24^,^25^,^49^. From the inspection of the PtVFX/2014 accessory genome for either known genomic islands or strain-specific genetic traits, we highlight the presence of an ~37.5 kb region that was found to be intact and highly similar (BLASTn, cover 100%, identity 99%, E-value 0.0) in only one strain (ATCC 33761 = DSM 21215) of *L. oakridgensis*, a species that rarely causes LD^50^. The SNP density analysis throughout the core-genome alignment of *L. pneumophila* PTVFX/2014 *versus L. oakridgensis* ATCC 33761 (DSM 21215) (Fig. 4A) confirms the similarity, and clearly points that this region was acquired by inter-species HGT. It carries genes potentially involved in conjugation, such as a *lvh/lvr* cluster coding for a typical T4SS (T4ASS) and some *tra* conjugal transfer genes^26^,^36^,^51^, as well as a gene cluster homolog to a type I-like restriction-modification system. While the *lvh/lvr* T4ASS is thought to be involved in *L. pneumophila* entry, delay of phagosome acidification and intracellular multiplication, which are virulence-related mechanisms that may boost its transition from a environmental bacterium to a human pathogen^52^, little is known about the role of the restriction-modification systems in *L. pneumophila*, although they have been suggested to be involved in its adaptation to different environmental niches^32^,^53^. Noteworthy, this ~37.5 kb genomic island was not found to be intact in any *L. pneumophila* strain with the genome available at the GenBank (including the closest related *L. pneumophila* subsp. *fraseri* ATCC33216/Dallas 1E strain). In fact, BLASTn analyses of this island only revealed partial homology (cover < 67%, identity < 98%, E-value 0.0) mainly targeting the region involving the T4ASS *lvh/lvr* cluster in other *L. pneumophila* strains. However, this might be explained by the presence of T4ASS *lvh/lvr* cluster integrated in either other genomic islands or genome locations. This is not surprising as *L. pneumophila* genomes may carry more than one *lvh/lvr* T4ASS cluster^27^,^30^,^31^,^36^,^49^. Indeed, for instance, PtVFX2014 strain possesses an additional *lvh/lvr* T4ASS cluster upstream to this ~37.5 kb genomic island. Interestingly, in close proximity of this additional *lvh/lvr* cluster, we detected three genes (*PtVFX2014_08925*/*cas1*, *PtVFX2014_08930*/*cas2* and *PtVFX2014_08930*/*cas4*) associated with the clustered regularly interspaced short palindromic repeats (CRISPR) system (CRISPR-associated genes – *cas*). The CRISPR-Cas system is a RNA-mediated immunity system used by some bacteria to resist to phages and other invading DNA^54^. In *L. pneumophila*, although little is known about the impact of the CRISPR system on regulation of virulence-related traits^55^, it has been demonstrated that the CRISPR-associated gene *cas2* of *L. pneumophila* strain 130b is required for intracellular infection of amoebae, indicating that it might play a role in the environment persistence and transmission of LD^56^. Of note, *cas* genes in the PTVFX/2014 strain resemble those annotated in the *L. pneumophila* strains 130 b and Paris^26^,^27^, but not in the 2300/99 Alcoy and Lens strains^55^.

Another relevant feature of PtVFX/2014 is the presence of a ~65 kb pathogenicity island that was previously reported to be absent in the most worldwide studied *L. pneumophila* strains^24^,^57^, with exception of the Philadelphia-1 strain, in which it was firstly found^58^. Like above, this PtVFX/2014 island was likely imported via HGT, being highly similar (Fig. 4B) to the one described in the Philadelphia-1 strain^58^. This island was recently found to mediate oxidative stress resistance *in vitro* and in macrophages^57^, carrying a plasmid F-like element comprising homologs of the T4SS (*tra/trb* genes), a *prpA-lvrABC* gene cluster (*PtVFX2014_07415*-*PtVFX2014_07430*), several membrane transporters, and multiple other potential virulence factors^58^. For instance, it harbors a homolog of an efflux pump YdhE/NorM belonging to the multidrug and toxin extrusion (MATE) family (*PtVFX2014_07605*), which has been associated with resistance to several antimicrobial agents in other bacteria^59^, and a gene encoding a macrophage infectivity potentiator-related protein (*lpg2112*/*PtVFX2014_07340*)^32^,^60^. The *prpA-lvrABC* gene cluster includes a *csrA* paralog (*lpg2094*/*PtVFX2014_07430*) encoding a RNA-binding protein that belongs to a superfamily of global regulators that are widely distributed among bacterial species. In *L. pneumophila*, CsrA-like proteins have been implicated in several mechanisms, including the bacterial switch to a resilient state yielding persistence in water supplies or the regulation of the transition between the replicative and transmissive phases of its pathogenic life cycle^46^,^61^,^62^,^63^. In conclusion, similarly to the outbreak-related Philadelphia-1 strain, the presence of this island endows PtVFX/2014 with machinery for excision and transfer as well as with a number of putative virulence factors and detoxifying enzymes that are believed to contribute to the emergence and persistence of best-fitted strains in natural or human-built water reservoirs and in human macrophages^57^,^58^.

### Repertoire of Dot/Icm T4BSS substrates in PtVFX/2014 strain

*L. pneumophila* encodes over 300 Dot/Icm T4BSS effectors that are translocated during the bacterial life-cycle into the host cell to subvert cellular functions, and thus, assuring intracellular survival and replication^47^,^49^,^64^,^65^. For instance, Dot/Icm effectors have been implicated in the bacterial uptake into the host cells, evasion of lysosome fusion, recruitment of host vesicles, lipid remodeling of the *Legionella*-containing vacuole, modulation of important cellular pathways (such as the host ubiquitin pathways) or host cell exit^29^,^66^,^67^,^68^. The majority of Dot/Icm effectors are shared by most *L. pneumophila* strains sequenced so far^47^,^49^,^64^,^65^. Nevertheless, there are still a considerable number of effectors that have been suggested to be present in just few strains or even to be strain-specific, making the investigation of the repertoire of Dot/Icm T4BSS effectors encoded in novel sequenced genomes important to understand potential strain-specific virulence or transmission traits.

In this regard, we screened the PtVFX/2014 genome for the presence/absence of 303 known Dot/Icm substrates coding genes that were recently gathered in the literature for six sequenced *L. pneumophila* strains (Philadelphia-1, 2300/99 Alcoy, Corby, Paris, Lens and 130b)^49^,^64^. This kind of presence/absence analysis has been mostly based on pairwise comparisons with data from the *L. pneumophila* Philadelphia-1 strain, so potential Dot/Icm effectors identified for the other sequenced strains were also not taken into account in the current study. Overall, 232 out of the 303 Dot/Icm substrates (~77%) are shared by the seven strains (Supplementary Table S2), which corroborates previous comparisons^47^,^49^,^64^,^65^. This observation gains even more relevance, since PVFX/2014 belongs to *L. pneumophila* subsp. *fraseri*, being phylogenetically distant from the strains used so far for Dot/Icm make-up comparisons. Thus, it is tempting to speculate that this set of ~230 shared substrates may constitute the “core” Dot/Icm arsenal within the *L. pneumophila* species.

**Figure 5:**
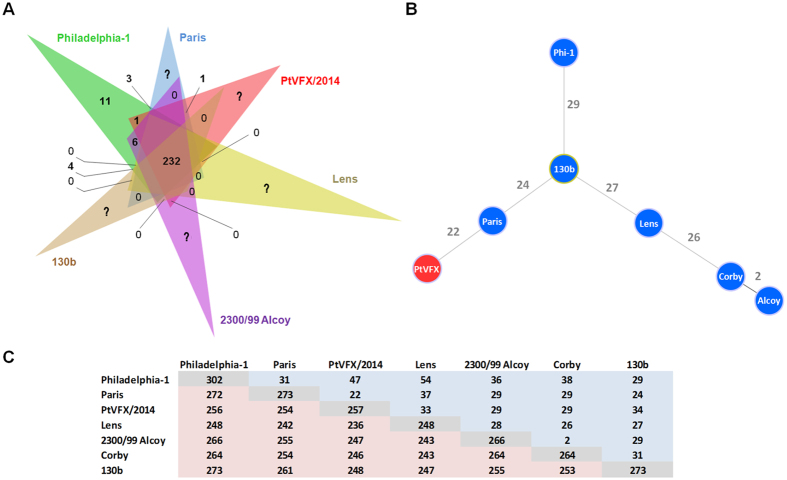
Repertoire of Dot/Icm T4BSS substrates in PtVFX/2014 strain. (**A**) Venn diagram [constructed using *jvenn*] showing the distribution of 303 known Dot/Icm substrates among six *L. pneumophila* strains. Given that any counting of strain-specific substrates would be an underestimation (as deep functional studies have not been systematically performed for all strains), those substrates were labeled with a question mark, except for the Philadelphia-1 strain as it was used as a query in the pairwise comparisons. Corby strain is not represented in the Venn diagram as it possesses a Dot/Icm repertoire almost identical to that of Alcoy. The only exceptions occur in the genes *lpg0080* and *lpg1965*, which are absent in Corby (*lpg0080* is also absent in Lens, whereas *lpg1965* is present in 130b and Philadelphia-1 besides 2300/99 Alcoy). Details regarding the Dot/Icm make-up of each strain are given in Supplementary Table S2. (**B**) goeBURST full minimum spanning tree showing that PtVFX/2014 (in red) carries a unique the Dot/Icm repertoire. Relationships based on the Dot/Icm make-up among strains were estimated using the goeBURST algorithm implemented in the PHYLOViZ software. Connecting lines are labeled with the number of Dot/Icm substrates differences between each strain. (**C**) Pairwise matrix displaying the number of Dot/Icm substrates that are shared (light red) or divergent (light blue) among strains. Diagonal values (in grey) represent the total number of Dot/Icm substrates inferred for each strain.

Regarding PtVFX/2014, we identified 257 known Dot/Icm substrates (Supplementary Table S2), whose repertoire is most similar to the one carried by the *L. pneumophila* Paris strain (Fig. 5), differing by only 8% (22/276) of the substrates. In particular, PtVFX/2014 exclusively shares the Dot/Icm effector coding gene *lpp2486*/*PtVFX2014_00550* with the Paris strain, which encodes an eukaryotic-like protein with an N-terminal F-box domain and a C-terminal coiled-coil motif that may play an important role in infection of *Acanthamoeba castellanii* and human macrophages^69^. Besides this substrate, we found just another Dot/Icm exclusively shared by PtVFX/2014 and a single strain, namely the gene *PtVFX2014_12325* homolog of the *lpg0402*/*ankY*/*legA9* of the Philadelphia-1 strain, which encodes a protein with an ankyrin motif. Despite its functional role is still unknown, ankyrin-containing proteins are thought to be important for *L. pneumophila* infection of eukaryotic host cells^70^. Indeed, AnkY/LegA9 was suggested to be involved in autophagy uptake of *Legionella*-containing vacuole and avoidance of lysosomal fusion^71^.

We cannot discard that PTVFX/2014 carries additional Dot/Icm substrates as, for instance, deep analysis of the recently sequenced 130b strain revealed the presence of 10 novel effectors (not addressed in Supplementary Table S2)^27^, where four of which (*lpw_00581*, *lpw_16311*, *lpw_20091* and *lpw_25791*) are also encoded in the PtVFX/2014 genome. Moreover, we detected some PtVFX/2014-exclusive genes that are adjacent to known Dot/Icm coding genes. One of those examples refers to *PtVFX2014_10440* (coding for a hypothetical protein), which is flanked by the two *dot*/*icm* genes *lpg0171*/*legU1* and *lpg0172.* Whether these exclusive genes constitute novel *L. pneumophila* Dot/Icm substrates cannot be stated, although they may constitute potential targets for future functional studies, since *dot*/*icm* genes often appear in clusters spread throughout the genome. Similarly to what has been shown for other *L. pneumophila* strains, the novel outbreak-related PtVFX/2014 strain revealed a unique composition of Dot/Icm effectors that may underlie a distinct virulence profile.

Overall, in this study, we analyzed the genetic backbone of the PtVFX/2014 strain, which was associated with the first strong evidence of person-to-person transmission and with the second worldwide largest outbreak of LD so far. Comparative genomics revealed that PtVFX/2014 possesses a unique genomic make-up, marked by relevant HGT events enrolling multiple virulence factors, which endow it with specific traits that may impact its ability to adapt and persist in diverse environments, and, ultimately, to be transmitted and to cause human disease. It is our goal to apply phenotypic approaches to dissect, in near future, the transmission and virulence skills of PtVFX/2014 strain.

## Methods

### WGS for outbreak investigation

To investigate the genetic relatedness of ST1905 isolates and to confirm the source of LD outbreak occurred in Portugal, 10 clinical isolates and one environmental isolate obtained from an industrial cooling tower were selected for WGS. Briefly, high-quality genomic DNA samples from pure bacterial cultures of both the clinical and the environmental isolates were used to prepare Nextera XT Illumina libraries that were subjected to paired-end sequencing (2 × 150 bp) on a MiSeq system (Illumina Inc.), according to the manufacturer’s instructions, using a depth of coverage >100-fold for all samples. Obtained reads were subjected to quality assessment and improvement (using FastQC and FASTX tools), and further assembled using the Velvet version 1.2.10 (https://www.ebi.ac.uk/~zerbino/velvet/). The *de novo* assembly process was optimized using the VelvetOptimiser script version 2.2.5. To study the genetic relatedness between each clinical isolate and the environmental isolate, a pairwise strategy was used in order to maximize the extent of core-genome to be compared. Briefly, pairwise alignments of the draft genome sequences were performed using the progressive algorithm of Mauve software (version 2.3.1) (http://darlinglab.org/mauve/mauve.html), and core-alignments were extracted by keeping and concatenating regions where sequences aligned over at least 500 bp. Both DnaSP v5 (http://www.ub.edu/dnasp/) and MEGA5 (http://www.megasoftware.net/) software were then used to check potential nucleotide variants, with Tablet 1.14.04.10 (https://ics.hutton.ac.uk/tablet/) being further applied to exclude false positive polymorphisms.

### goeBURST analysis based on allelic profiles of *L. pneumophila* STs

The European Working Group for *Legionella* Infections (EWGLI) SBT database (http://www.ewgli.org/)^16^,^17^ included data of 10085 isolates (with ST attributed) from 60 countries representing 2108 distinct STs, at the time this analysis was conducted (24.11.2015). To display the relationships between the allelic profile of ST1905 and the other STs described worldwide, we constructed a goeBURST full minimum spanning tree (MST)^72^ implemented in the PHYLOViZ software^73^.

### Whole-genome comparative analyses within the *L. pneumophila* species

To integrate the genetic backbone of the *L. pneumophila* PtVFX/2014 strain in the frame of the species phylogeny, 10 close chromosome sequences (including those from the most studied outbreak-associated *L. pneumophila* serogroup 1 strains), four draft genomes from *L. pneumophila* subspecies other than the *L. pneumophila* subsp. *pneumophila* available at GenBank, and sequence data (retrieved from European Nucleotide Archive) from 10 isolates selected for increasing genetic diversity (see Supplementary Table S1) were also analyzed. This selection criterion allowed us to work with the 15 highly diverse phylogenetic clusters described by Underwood and colleagues^20^. A core-genome alignment (enrolling a total of 35 genome sequences) followed by phylogenetic inferences were performed with Parsnp implemented on Harvest suite^74^, using the default parameters, with exception of parameter –C, which was adjusted to 2000 in order to maximize the reference coverage. Whole-genome pairwise alignments performed using the progressive algorithm of MAUVE 2.3.1 software were also analyzed through the DnaSP v5 software in order to identify genomic regions with high single nucleotide polymorphism (SNP) density. SimPlot/BootScan software (http://sray.med.som.jhmi.edu/SCRoftware/simplot/) was used to precisely identify breakpoint regions of putative recombination events. Multiple additional MAUVE alignments, RAST (http://rast.nmpdr.org/) and BLAST analyses were also performed to characterize the genome of the outbreak-associated ST1905 strain in regard to the presence of specific genomic islands, plasmids, prophages, virulence factors, and other relevant features.

### Genetic analysis of wzt and wzm loci

In order to investigate the genetic basis determining the serogroup type of the strain PtVFX/2014 (serogroup 1), we analyzed the nucleotide sequences of two genes (*wzt* and *wzm*) belonging to the lipopolysaccaride (LPS) cluster of *L. pneumophila*^39^, since these loci were found to be good genetic markers for discriminating *L. pneumophila* serogroups^34^,^35^. In this regard, phylogenetic reconstructions over individual gene alignments were performed through MEGA5 by using the neighbor-joining method with bootstrapping (1000 replicates) and the Kimura-2-parameter for distance estimates.

### Nucleotide sequence accession number

A draft genome sequence of one representative clone of the outbreak-related ST1905 strain (PtVFX/2014) was submitted to GenBank, annotated using the NCBI Prokaryotic Genomes Annotation Pipeline 2.3, and is available under the accession number LORH00000000. Raw sequence reads of each PtVFX/2014 strain were deposited in Sequence Read Archive (SRA) (http://www.ncbi.nlm.nih.gov/sra) under the accession numbers: SRR3176625, SRR3176631, SRR3176714, SRR3176716, SRR3176831, SRR3176838, SRR3176884, SRR3176894, SRR3176900, SRR3176901 and SRR3176904.

## Additional Information

**How to cite this article**: Borges, V. *et al.* *Legionella pneumophila* strain associated with the first evidence of person-to-person transmission of Legionnaires’ disease: a unique mosaic genetic backbone. *Sci. Rep.* **6**, 26261; doi: 10.1038/srep26261 (2016).

## Supplementary Material

Supplementary Information

## Figures and Tables

**Table 1:** Description of HGT-inherited regions in PtVFX/2014.

Region	Estimated length (Kbp)	Genome location		Presence of Dot/Icm effectors coding genes^c^		Relevant genes included^a^,^d^
		*loci*Philadelphia-1 strain^a^	*loci* PtVFX/2014^b^	Philadelphia-1^a^	PtVFX/2014^b^	
A	113	*lpg0831-lpg0723*	PtVFX2014_03240-03730	*lpg0733*	PtVFX2014_03780	LPS biosynthesis loci (*lpg0748-lpg0779*)^e^ (VFDB; 39); *lpg0791*/*mip*^40^; The iron assimilation- and virulence-associated *lpg0746/iraAB locus* (VFDB^37^;); *lpg0742/ldsA*^38^
B	21	*lpg1025(yegE)-lpg0986*	PtVFX2014_08445-08505	—	—	Genes potentially associated with resistance to antibiotics and toxic compounds
C	25	*lpg1640-lpg1619(ftsl4)*	PtVFX2014_04280-04395	*lpg1639; lpg1625; lpg1621*	PtVFX2014_04285; PtVFX2014_04365; PtVFX2014_04385	MecI family transcriptional regulator associated with β-lactams resistance (*lpg1620*)
D	8	*lpg1794-lpg1800(recX)*	PtVFX2014_11245-11275	*lpg1798*	PtVFX2014_11260	Transcriptional regulator LysR (*lpg1796*); Regulatory protein RecX (*lpg1800*).
E	33	*lpg1817-lpg1845(vacJ)*	PtVFX2014_11360-11500	*lpg1822; lpg1836*	PtVFX2014_11385; PtVFX2014_11455	*lpg1845/vacJ*: encodes a lipoprotein associated with peptidoglycan stabilization (potential virulence factor in cell to cell migration^36^); *lpg1818/msbA*: encodes the Lipid A export ATP-binding/permease protein MsbA^41^
F	20	*lpg2282(asnS)-lpg2262(ackA2)*	PtVFX2014_01325-01425	*lpg2271*	PtVFX2014_01380	—
G	34	*lpg2462-lpg2426(mdcA)*	PtVFX2014_00340-00520	*lpg2461; lpg2456; lpg2452; lpg2444; lpg2443; lpg2434; lpg2433*	PtVFX2014_00345; PtVFX2014_00370; PtVFX2014_00395; PtVFX2014_00425; PtVFX2014_00430; PtVFX2014_00480; PtVFX2014_00485	*lpg2438:* encodes a florfenicol efflux pump
H	33	*lpg2815-lpg2842(phoH)*	PtVFX2014_14890-15010	*lpg2815; lpg2826; lpg2828; lpg2832*	PtVFX2014_14890; PtVFX2014_14945; PtVFX2014_14955; PtVFX2014_14960	*lpg2839/smpB* (gene likely essential for viability of *L. pneumophila*^42^); *lpg2825*: gene associated with cold shock; *lpg2841*: putative antibiotic resistance-associated gene.

^a^*Loci* designations are based on the Philadelphia-1 strain genome annotation (GenBank accession number AE017354).

^b^*Loci* designations are based on the PtVFX/2014 strain genome annotation (GenBank accession number LORH00000000) (this study).

^c^Presence/absence analysis based on 303 known Dot/Icm substrates coding genes that were recently gathered in the literature for six sequenced *L. pneumophila* strains (Philadelphia-1, 2300/99 Alcoy, Corby, Paris, Lens and 130b).

^d^Based on specific literature and/or on the Virulence Factors Database (VFDB) (http://www.mgc.ac.cn/VFs/).

^e^Correspond to the *loci* PtVFX2014_03470-03605. The genes *lpg0778* and *lpg0675* are absent in the PtVFX/2014 strain.
