# Supplementary material for: Legionella pneumophila strain associated with the first evidence of person-to-person transmission of Legionnaires’ disease: a unique mosaic genetic backbone
Source: Sci Rep. 2016 May 19;6:26261. doi: 10.1038/srep26261 (PMC4872527; doi:10.1038/srep26261)
Supplement: Supplementary Information [file srep26261-s1.pdf]

Legionella pneumophila strain associated with the first evidence of person-to-person transmission of Legionnaires' disease: a unique mosaic genetic backbone

Vítor Borges, Alexandra Nunes, Daniel A. Sampaio, Luís Vieira, Jorge Machado, Maria J. Simões, Paulo Gonçalves, and João P. Gomes

Supplementary Information

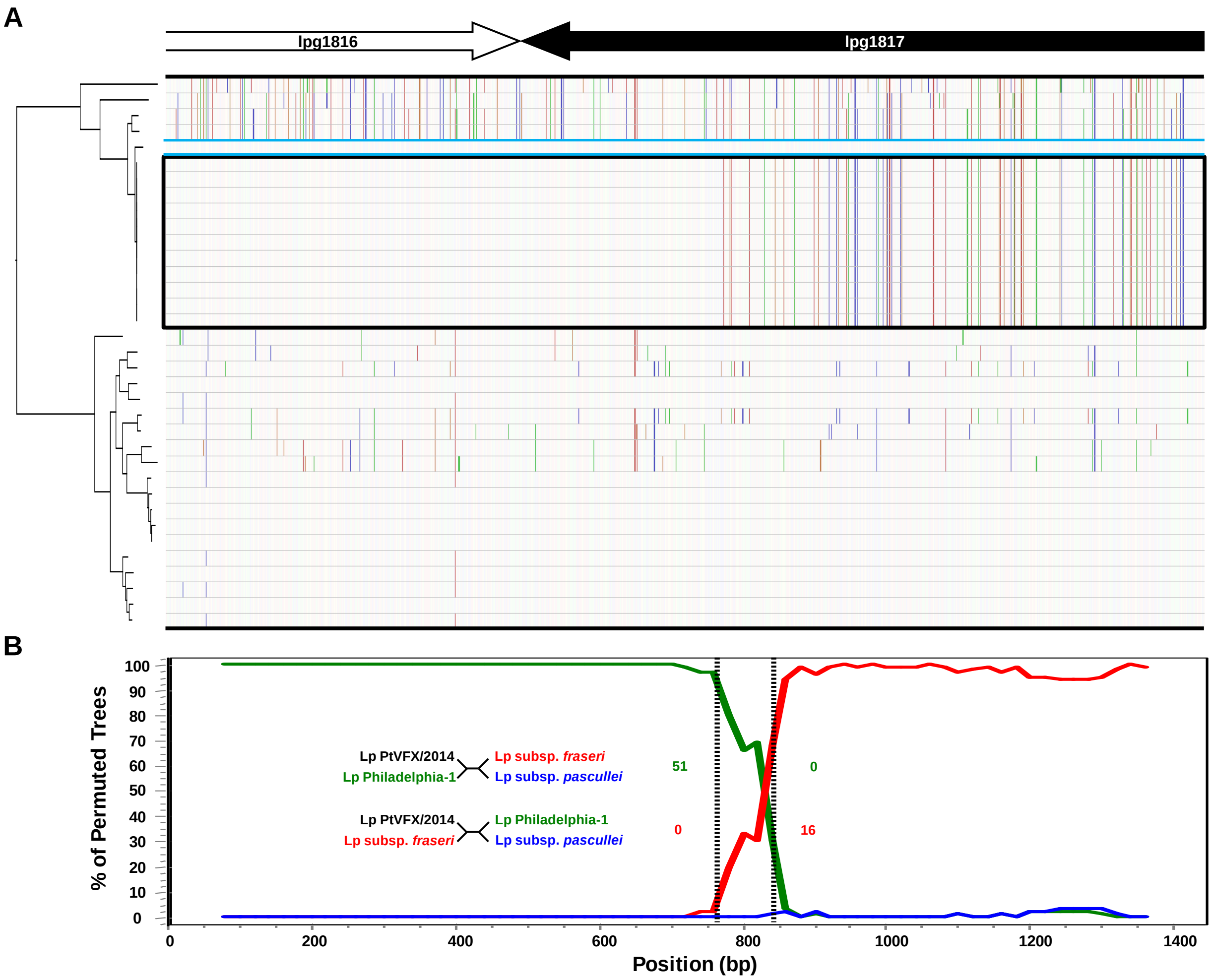

Supplementary Figure S1. Detailed analysis of a cross-over region in PtVFX/2014. (a) Zoomed visualization of the SNP density profile of Figure 2A highlighting a recombination breakpoint (boxed in black) that affects the gene *lpg1817* (black arrow) and involves the exchange of the region E (Fig. 2B and Table 1). The vertical lines represent SNPs against the genome of the *L. pneumophila* subsp. *fraseri* ATCC33216 strain (surrounded with a blue box). (b) BootScan analysis (window size 60 bp; step size 20 bp) showing the phylogenetic relatedness (% of permuted trees) between PtVFX/2014, the putative parental strains (Philadelphia-1 and *L. pneumophila* subsp. *fraseri* ATCC35251) and the outgroup strain (*L. pneumophila* subsp. *pascullei* ATCC33737). The crossover region is precisely located between the vertical black dashed lines and involves less than 80 bp. Fifty-one informative sites support the similarity between PtVFX/2014 and Philadelphia-1 (in green) in the left side of the breakpoint, whereas 16 support its similarity with *L. pneumophila* subsp. *pascullei* ATCC33737 (in red) ($P < 10^{-7}$; maximum $\chi 2$ test) in the right side of the breakpoint. In these defined regions there are no informative sites supporting the alternative hypotheses.

Supplementary Table S1. List of *Legionella* spp. strains used for comparative genomic analyses.

Strain name	Serogroup [a]	Sequence type (ST) [b]	Sequence accession number	References
L. pneumophila PtVFX/2104	1	1905	LORH00000000 [c]	This study
L. pneumophila subsp. *pneumophila* Philadelphia-1	1	36	AE017354.1 [c]	[1-2]
L. pneumophila Paris	1	1	CR628336.1 [c]	[2-4]
L. pneumophila Lens	1	15	CR628337.1 [c]	[2-4]
L. pneumophila Corby	1	51	CP000675.2 [c]	[2,4-5]
L. pneumophila subsp. *pneumophila* Lorraine	1	47	FQ958210.1 [c]	[2,6-7]
L. pneumophila 2300/99 Alcoy	1	578	CP001828.1 [c]	[2,4,8]
L. pneumophila 130b (Wadsworth)	1	42	CAFM01000001-CAFM01000159 [c,d] / FR687201 [c]	[2,9]
L. pneumophila subsp. *pneumophila* HL06041035	1	734	FQ958211.1 [c]	[10]
L. pneumophila subsp. *pneumophila* ATCC 43290 (or 570-CO-H)	12	187	CP003192.1 [c]	[11-12]
L. pneumophila subsp. *pneumophila*ThunderBay	6	187	CP003730.1 [c]	[4]
L. pneumophila subsp. *pneumophila* LPE509	1	780-like	CP003885.1 [c]	[13]
L. pneumophila H093620212	1	46	ERR315646 [d]	[2]
L. pneumophila H090500162	1	611	ERR315652 [d]	[2]
L. pneumophila LC6677	3	87	ERR315653 [d]	[2]
L. pneumophila RR08000517	9	337	ERR315658 [d]	[2]
L. pneumophila H063280001	1	23	ERR315663 [d]	[2]
L. pneumophila H091960011	1	454	ERR315665 [d]	[2]
L. pneumophila H070840415	1	59	ERR315666 [d]	[2]
L. pneumophila RR08000134	1	34	ERR315670 [d]	[2]
L. pneumophila H091960009	4	707	ERR315672 [d]	[2]
L. pneumophila subsp. *fraseri* ATCC 33216 (or Dallas 1E)	5	1300	GCA_000586275.1 [e]	[11]
L. pneumophila subsp. *fraseri* ATCC 33156 (or Los Angeles-1)	4	1334	GCA_000586315.1 [e]	[11]

L. pneumophila subsp. *fraseri* ATCC 35251 (or Lansing-3)	15	336	GCA_000586195.1 [e]	11
L. pneumophila subsp. *pascullei* ATCC 33737 (or U8W)	5	1335	GCA_000586255.1 [e]	14
L. oakridgensis ATCC 33761 (or DSM 21215)	---	---	CP004006 [c]	15

[a] Serogroup numbers were obtained from the European Working Group for *Legionella* Infections (EWGLI) sequence-based typing (SBT) database[16-18] or from the literature. The serogroup number of the strain LC6677 (serogroup 3) refers to the one described by Underwood and colleagues[2] (also confirmed through the *wzt*/*wzm*-based *in silico* analysis)[19-20], since it is wrongly fulfilled in the EWGLI database. The strain RR08000517 was considered to belong to serogroup 9 (and not to the serogroup 4, as fulfilled in the EWGLI database), since *in silico* analyses based on the *wzt*/*wzm*-based scheme revealed that the *wzt*/*wzm* sequences matched the serogroup 9 reference sequence, strongly suggesting that the attribution of serogoup 4 in the EWGLI is likely an error.

[b] Sequence types (STs) were retrieved from the EWGLI SBT database[16-18] or extracted/confirmed *in silico*. No ST number is attributed to the strain LPE509, but its allelic profile differ by just one allele (alllele 1 in locus *mompS*) from ST780. The ST number of the strain LC6677 (ST87) refers to the one described by Underwood and colleagues[2] (also confirmed *in silico*), since its ST attribution is wrongly filled in the EWGLI database.

[c] Numbers refer to the Nucleotide GenBank database (NCBI).

[d] Numbers refer to the European Nucleotide Archive (ENA) database.

[e] Numbers refer to the GenBank Assembly database (NCBI).

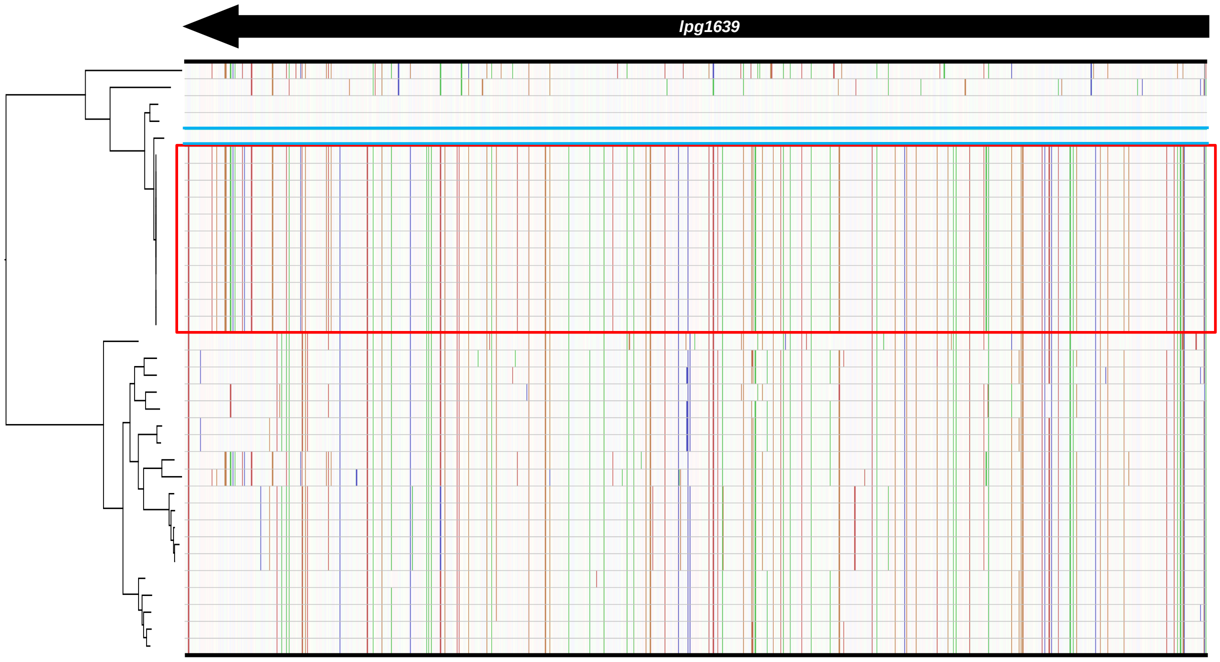

Supplementary Figure S2. Mutational dynamics of the Dot/Icm substrate coding gene *lpg1639*. Zoomed visualization of the SNP density profile within the HGT-acquired region C of Figure 2, with vertical lines representing SNPs against the genome of *L. pneumophila* subsp. *fraseri* ATCC33216 strain (surrounded with a blue box). This figure underlines the huge concentration of homoplasic mutations shared between the PtVFX/2014 clones (boxed in red) and the most worldwide studied outbreak-associated *L. pneumophila* serogroup 1 strains.

Supplementary Table S2. Repertoire of Dot/Icm substrates of the *L. pneumophila* PtVFX/2014 strain.

strain [a, b]							
**Philadelphia-1**	**Paris**	**Lens**	**Corby**	**2300/99 Alcoy**	**130b**	**PtVFX/2014**	**Alternative gene designation**
lpg0008	lpp0008	lpl0008	lpc0009	lpa0011	lpw00071	PtVFX2014_09980	*ravA*
lpg0012	lpp0012	lpl0012	lpc0013	lpa0016	lpw00111	PtVFX2014_09960	*cegC1*
lpg0021	lpp0021	lpl0022	lpc0022	lpa0030	lpw00221	PtVFX2014_09915	*ND*
lpg0030	lpp0030	lpl0031	lpc0031	lpa0040	lpw00311	PtVFX2014_09870	*ravB*
lpg0038	lpp0037	lpl0038	lpc0039	lpa0049	lpw00381	PtVFX2014_09835	*ankQ/legA10*
lpg0041	–	–	lpc0042	lpa0056	–	–	*ND*
lpg0045	lpp0046	lpl0044	lpc0047	lpa0060	lpw00441	PtVFX2014_09795	*ND*
lpg0046	lpp0047	lpl0045	lpc0048	lpa0062	lpw00451	PtVFX2014_09790	*ND*
lpg0059	lpp0062	lpl0061	lpc0068	lpa0085	lpw00621	PtVFX2014_09705	*ceg2*
lpg0080	lpp0094	–	–	lpa3018	lpw00781	PtVFX2014_09590	*ceg3*
lpg0081	lpp0095	–	–	–	lpw00791	PtVFX2014_09585	*ND*
lpg0090	lpp0104	lpl0089	lpc0109	lpa0132	lpw00881	PtVFX2014_09540	*lem1*
lpg0096	lpp0110	lpl0096	lpc0115	lpa0145	lpw00961	PtVFX2014_09505	*ceg4*
lpg0103	lpp0117	lpl0103	lpc0122	lpa0152	lpw01031	PtVFX2014_09470	*vipF*
lpg0107	lpp0121	lpl0107	lpc0126	lpa0159	lpw01071	PtVFX2014_09450	*ravC*
lpg0126	lpp0140	lpl0125	lpc0146	lpa0185	lpw01261	PtVFX2014_09360	*cegC2*
lpg0130	lpp0145	lpl0130	lpc0151	lpa0194	lpw01311	PtVFX2014_09340	*ND*
lpg0135	lpp0150	lpl0135	lpc0156	lpa0204	lpw01361	PtVFX2014_09315	*sdhB*
lpg0140	lpp0155	lpl0140	lpc0161	lpa0214	lpw01411	PtVFX2014_09290	*cetLP1*
lpg0160	lpp0224	lpl0224	lpc0242	lpa0322	lpw02541	PtVFX2014_10380	*ravD*
lpg0170	lpp0232	lpl0233	lpc0251	lpa0335	lpw02641	PtVFX2014_10430	*ravC*
lpg0171	lpp0233	lpl0234	Ψ ?	Ψ ?	lpw02651	PtVFX2014_10435	*legU1*
lpg0172	lpp0234	–	lpc0253	lpa0339	lpw02661	PtVFX2014_10445	*ND*
lpg0181	lpp0245	lpl0244	lpc0265	lpa0388	lpw02761	PtVFX2014_10495	*ND*
lpg0191	lpp0251	–	–	–	lpw02821	–	*ceg5*
lpg0195	lpp0253	lpl0251	lpc0272	lpa0339	lpw02851	PtVFX2014_10555	*ravE*
lpg0196	lpp0254	lpl0252	–	–	lpw02861	PtVFX2014_10560	*ravF*
lpg0208	lpp0267	lpl0262	lpc0283	lpa0386	lpw02961	PtVFX2014_10615	*ceg6/legK4*

lpg0209	lpp0268	lpl0263	lpc0284	lpa0387	lpw02971	PtVFX2014_10620	*mavR*
lpg0210	lpp0269	lpl0264	lpc0285	lpa0388	lpw02981	PtVFX2014_10625	*ravG*
lpg0227	lpp0286	lpl0281	lpc0303	lpa0412	lpw03151	PtVFX2014_10710	*ceg7*
lpg0234	lpp0304	lpl0288	lpc0309	lpa0419	lpw03221	PtVFX2014_10745	*sidE/laiD*
lpg0240	lpp0310	lpl0294	lpc0316	lpa0428	lpw03291	PtVFX2014_10775	*ceg8*
lpg0246	lpp0316	lpl0300	lpc0323	lpa0436	lpw03361	Ψ ?	*ceg9*
lpg0254	lpp0324	lpl0307	lpc0331	lpa0446	lpw03431	PtVFX2014_10845	*pelA*
lpg0257	lpp0327	lpl0310	lpc0334	lpa0450	lpw03461	PtVFX2014_10860	*sdeA*
lpg0260	lpp0332	lpl0313	lpc0337	lpa0456	lpw03491	PtVFX2014_10875	*ND*
lpg0275	lpp0349	lpl0327	lpc0351/3529	lpa0477	lpw03641	PtVFX2014_13370	*sdbA*
lpg0276	lpp0350	lpl0328	lpc0353	lpa0479	lpw03651	PtVFX2014_13375	*legG2*
lpg0284	lpp0360	lpl0336	lpc0361	lpa0490	lpw03741	PtVFX2014_13415	*ceg10*
lpg0285	lpp0361	lpl0337	lpc0362	lpa0492	lpw03751	PtVFX2014_13420	*lem2*
lpg0294	lpp0372	lpl0347	lpc0373	lpa0508	lpw03861	PtVFX2014_13470	*ND*
lpg0364	lpp0429	lpl0405	lpc2980	lpa0578	lpw04431	PtVFX2014_12140	*ND*
lpg0365	lpp0430	lpl0406	lpc2979	lpa0580	lpw04441	PtVFX2014_12145	*ND*
lpg0375	lpp0442	lpl0418	lpc2968	lpa0596	Ψ (lpw04581)	PtVFX2014_12195	*ND*
lpg0376	lpp0443	lpl0419	lpc2967	lpa0597	lpw04591	PtVFX2014_12200	*sdhA*
lpg0390	lpp0457	lpl0433	lpc2954	lpa0613	lpw04721	PtVFX2014_12265	*vipA*
lpg0393	lpp0461	lpl0437	lpc2949	lpa0619	lpw04761	PtVFX2014_12285	*cetLP2*
lpg0401	lpp0468	lpl0444	lpc2942	lpa0629	lpw04831	PtVFX2014_12320	*legA7/ceg11*
lpg0402	–	–	–	–	–	PtVFX2014_12325	*ankY/legA9*
lpg0403	lpp0469	lpl0445	lpc2941	lpa0630	lpw04841	PtVFX2014_12330	*ankG/ankZ/legA7*
lpg0405	lpp0471	lpl0447	lpc2939	lpa0633	lpw04861	PtVFX2014_12340	*ND*
lpg0422	lpp0489	lpl0465	lpc2921	lpa0657	lpw05041	PtVFX2014_12430	*legY*
lpg0436	lpp0503	lpl0479	lpc2906	lpa0673	lpw05181	PtVFX2014_12500	*ankJ/legA11*
lpg0437	lpp0504	lpl0480	lpc2905	lpa0674	lpw05191	PtVFX2014_12505	*ceg14*
lpg0439	lpp0505	lpl0481	lpc2904	lpa0678	lpw05201	PtVFX2014_12510	*ceg15*
lpg0483	lpp0547	lpl0523	lpc2861	lpa0739	lpw05631	PtVFX2014_12720	*ankC/legA12*
lpg0515	lpp0578	lpl0554	lpc2829	lpa0776	lpw05951	–	*legD2*
lpg0518	lpp0581	lpl0557	lpc2826	lpa0781	lpw05981	Ψ ?	*ND*
lpg0519	–	–	–	–	–	–	*ceg17*

lpg0621	lpp0675	lpl0658	lpc2673	lpa0975	lpw06951	PtVFX2014_13870	*sidA*
lpg0634	lpp0688	lpl0671	lpc2660	lpa0996	lpw07081	PtVFX2014_13805	*ND*
lpg0642	lpp0696	lpl0679	lpc2651	lpa1005	lpw07161	PtVFX2014_13765	*wipB*
lpg0695	lpp0750	lpl0732	lpc2599	lpa1082	lpw07721	PtVFX2014_03870	*ankN/ankX/legA8*
lpg0696	lpp0751	lpl0733	lpc2598	lpa1084	lpw07731	PtVFX2014_03865	*lem3*
lpg0716	lpp0782	lpl0753	lpc2577	lpa1108	lpw07931	PtVFX2014_03765	*ND*
lpg0733	lpp0799	lpl0770	lpc2559	lpa1135	lpw08111	PtVFX2014_03780	*ravH*
lpg0796	lpp0859	–	–	–	–	–	*ND*
lpg0898	lpp0959	lpl0929	lpc2395	lpa1360	lpw09801	PtVFX2014_02870	*ceg18*
lpg0921	lpp0982	lpl0952	lpc2370	lpa1391	lpw10051	PtVFX2014_02755	*mavT*
lpg0926	lpp0988	lpl0957	lpc2365	lpa1397	lpw10111	PtVFX2014_02730	*ravI*
lpg0940	lpp1002	lpl0971	lpc2349	lpa1415	lpw10251	PtVFX2014_02660	*lidA*
lpg0944	lpp1006	–	lpc2345	lpa1421	lpw10301	PtVFX2014_02640	*ravJ*
lpg0945	lpp1007	lpl1579	lpc2344	lpa1423	lpw10311	PtVFX2014_02635	*legL1*
lpg0963	lpp1025	lpl0992	lpc2324	lpa1453	lpw10491	PtVFX2014_02545	*ND*
lpg0967	lpp1029	Ψ (lpl0996a/b)	lpc2320	lpa1459	lpw10531	PtVFX2014_02525	*ND*
lpg0968	lpp1030	lpl0997	lpc2319	lpa1460	lpw10541	PtVFX2014_02520	*sidK*
lpg0969	lpp1031	lpl0998	lpc2318	lpa1461	lpw10551	PtVFX2014_02515	*ravK*
lpg1083	–	–	–	–	–	–	*ND*
lpg1101	lpp1101	lpl1100	# (lpc2154)	# (lpa1709)	lpw11451	–	*lem4*
lpg1106	lpp1105	lpl1105	lpc2149	lpa1719	lpw11501	PtVFX2014_02395	*ND*
lpg1108	lpp1108	lpl1108	lpc2146	lpa1724	lpw11531	PtVFX2014_02385	*ravL*
lpg1109	lpp1109	– $	lpc2145	lpa1725	Ψ ? (lpw11541/ lpw111551)	PtVFX2014_02380	*ravM*
lpg1110	lpp1111	lpl1114	lpc2142	lpa1728	lpw11571	PtVFX2014_02370	*lem5*
lpg1111	lpp1112	lpl1115	lpc2141	lpa1730	lpw11581	PtVFX2014_02365	*ravN*
lpg1120	–	–	–	–	lpw11681	–	*lem6*
lpg1121	lpp1121	lpl1126	lpc0578	lpa1743	lpw11691	PtVFX2014_02320	*ceg19*
lpg1124	lpp1125	lpl1129	lpc0582	lpa1748	lpw11741	PtVFX2014_02295	*ND*
lpg1129	lpp1130	–	– $	– $	lpw11801	PtVFX2014_02275	*ravO*
lpg1137	lpp1139	lpl1144	lpc0601	lpa1776	lpw11901	PtVFX2014_02230	*ND*

lpg1144	lpp1146	lpl1150	lpc0607	lpa1785	lpw11971	PtVFX2014_02200	*cegC3*
lpg1145	lpp1147	lpl1151	lpc0608	lpa1787	lpw11981	PtVFX2014_02195	*lem7*
lpg1147	lpp1149	lpl1153	lpc0610	lpa1789	lpw12001	PtVFX2014_02185	*ND*
lpg1148	lpp1150	lpl1154	lpc0611	lpa1790	lpw12011	PtVFX2014_02180	*ND*
lpg1152	lpp1154	lpl1159	lpc0615	lpa1795	lpw12061	PtVFX2014_02160	*ravP*
lpg1154	lpp1156	lpl1161	lpc0617	lpa1797	lpw12081	PtVFX2014_02140	*ravQ*
lpg1158	lpp1160	# (lpl1165)	lpc0621	lpa1802	lpw12121	PtVFX2014_02120	*ND*
lpg1166	lpp1168	lpl1174	lpc0631	lpa1819	lpw12211	PtVFX2014_02080	*ravR*
lpg1171	lpp1173	lpl1179	lpc0637	lpa1826	lpw12261	PtVFX2014_02055	*ND*
lpg1183	lpp1186	lpl1192	lpc0650	lpa1839	lpw12401	PtVFX2014_01995	*ravS*
lpg1227	lpp1235	lpl1235	lpc0696	lpa1899	lpw12861	PtVFX2014_06245	*vpdB*
lpg1273	lpp1236	lpl1236	lpc0698	lpa1901	lpw12871	PtVFX2014_06145	*ND*
lpg1290	lpp1253	–	–	–	–	PtVFX2014_06060	*lem8*
lpg1312	–	–	–	–	lpw13261	–	*legC1*
lpg1316	–	–	–	–	–	–	*ravT*
lpg1317	–	–	–	–	–	–	*ravW*
lpg1328	lpp1283	lpl1282	lpc0743	lpa1958	Ψ (lpw00991)	PtVFX2014_05910	*legT*
lpg1354	lpp1308	–	–	–	–	Ψ ?	*ND*
lpg1355	lpp1309	–	–	–	–	Ψ ?	*sidG*
lpg1356	lpp1310	lpl1307	lpc0770	lpa1998	lpw13631	PtVFX2014_05770	*ND*
lpg1368	lpp1322	lpl1319	lpc0784	lpa2017	lpw13751	Ψ ?	*lgt1*
lpg1408	lpp1363	lpl1359	lpc0824	lpa2071	Ψ ? (lpw14211/ lpw14221)	PtVFX2014_05480	*licA*
lpg1426	lpp1381	lpl1377	lpc0842	lpa2090	lpw14431	PtVFX2014_05390	*vpdC*
lpg1449	lpp1404	–	–	–	lpw14671	PtVFX2014_05275	*ND*
lpg1453	lpp1409	lpl1591	lpc0868	lpa2119	lpw14711	PtVFX2014_05255	*ND*
lpg1483	lpp1439	lpl1545	lpc0898	lpa2161	lpw15031	PtVFX2014_05110	*legK1*
lpg1484	lpp1440	lpl1544	lpc0899	lpa2162	lpw15041	PtVFX2014_05105	*ND*
lpg1488	lpp1444	lpl1540	lpc0903	lpa2168	lpw15081	PtVFX2014_05085	*lgt3/legc5*
lpg1489	lpp1445	lpl1539	lpc0905	lpa2169	lpw15091	PtVFX2014_05080	*ravX*
lpg1491	lpp1447	–	–	–	–	–	*lem9*
lpg1496	lpp1453	lpl1530	lpc0915	lpa2185	lpw15181	PtVFX2014_05030	*lem10*

lpg1551	lpp1508	lpl1475	lpc0972	lpa2253	lpw15731	PtVFX2014_04755	*ravY*
lpg1578	lpp4178	lpl4143	lpc1002	lpa2292	lpw16011	PtVFX2014_04615	*ND*
lpg1588	lpp1546	lpl1437	lpc1013	lpa2305	lpw16131	PtVFX2014_04560	*legC6*
lpg1598	lpp1556	lpl1427	lpc1025	lpa2317	lpw16231	PtVFX2014_04510	*lem11*
lpg1602	lpp1567	– $	lpc1028	lpa2318	lpw16241	–	*legL2*
lpg1621	lpp1591	lpl1402	lpc1048	lpa2346	lpw16461	PtVFX2014_04385	*ceg23*
lpg1625	lpp1595	lpl1398	lpc1052	lpa2350	lpw16511	PtVFX2014_04365	*lem23*
lpg1639	lpp1609	lpl1387	lpc1068	lpa2367	lpw16651	PtVFX2014_04285	*ND*
lpg1642	Ψ	lpl1384	lpc1071	lpa2371	lpw16681	–	*sidB*
lpg1654	lpp1625	–	lpc1084	lpa2390	–	PtVFX2014_04215	*ND*
lpg1660	lpp1631	lpl1625	lpc1090	lpa2398	lpw16861	PtVFX2014_04185	*legL3*
lpg1661	lpp1632	lpl1626	lpc1091	lpa2399	lpw16871	PtVFX2014_04180	*ND*
lpg1663	lpp1634	lpl1628	lpc1093	lpa2402	lpw16891	PtVFX2014_04170	*cetLP3*
lpg1666	lpp1637	lpl1631	lpc1096	lpa2408	lpw16921	PtVFX2014_04155	*ND*
lpg1667	lpp1638	lpl1632	lpc1097	lpa2409	lpw16931	PtVFX2014_04150	*ND*
lpg1670	lpp1642	lpl1635	lpc1101	lpa2413	lpw16971	PtVFX2014_04130	*ND*
lpg1683	–	–	lpc1114	lpa2431	–	–	*ravZ*
lpg1684	–	–	lpc1115	lpa2432	–	–	*ND*
lpg1685	–	–	lpc1116	lpa2433	–	–	*ND*
lpg1687	lpp1656	lpl1650	lpc1118	lpa2437	lpw17121	PtVFX2014_04055	*mavA*
lpg1689	lpp1658	lpl1652	lpc1120	lpa2439	lpw17141	PtVFX2014_04045	*ND*
lpg1692	–	–	lpc1123	lpa2442	–	–	*ND*
lpg1701	lpp1666	lpl1660	lpc1130	lpa2455	lpw17231	PtVFX2014_04000	*ppeA/legC3*
lpg1702	lpp1667	lpl1661	lpc1131	lpa2456	lpw17241	PtVFX2014_03995	*ppeB*
lpg1716	lpp1681	lpl1675	lpc1146	lpa2474	lpw17391	PtVFX2014_03925	*ND*
lpg1717	lpp1682	–	–	–	lpw17401	PtVFX2014_03920	*ND*
lpg1718	lpp1683	lpl1682	lpc1152	lpa2484	lpw17411	PtVFX2014_03915	*ankI/legAS4*
lpg1751	lpp1715	lpl1715	lpc1191	lpa2538	lpw17761	PtVFX2014_11030	*ND*
lpg1752	lpp1716	lpl1716	lpc1192	lpa2539	lpw17771	PtVFX2014_11035	*ND*
lpg1776	lpp1740	lpl1740	lpc1217	lpa2570	lpw18031	PtVFX2014_11155	*ND*
lpg1797	–	–	lpc1239	lpa2599	lpw32931	–	*rvfA*
lpg1798	lpp1761	lpl1761	lpc1241	lpa2600	lpw18281	PtVFX2014_11260	*marB*

lpg1803	lpp1766	lpl1766	lpc1246	lpa2606	lpw18331	PtVFX2014_11285	*ND*
lpg1822	lpp1785	lpl1786	lpc1266	lpa2634	lpw18531	PtVFX2014_11385	*cetLP4*
lpg1836	lpp1799	lpl1800	lpc1280	lpa2652	lpw18691	PtVFX2014_11455	*ceg25*
lpg1851	lpp1818	lpl1817	lpc1296	lpa2675	lpw18871	PtVFX2014_11535	*lem14*
lpg1884	lpp1848	lpl1845	lpc1331	lpa2714	lpw19161	PtVFX2014_11715	*ylfB/legC2*
lpg1888	lpp1855	lpl1850	lpc1336	lpa2723	lpw19211	PtVFX2014_11745	*ND*
lpg1890	lpp1857	lpl1852	lpc1338	lpa2726	lpw19231	PtVFX2014_11755	*legLC8*
lpg1907	lpp1882	lpl1871	lpc1361	lpa2762	lpw19461	PtVFX2014_11840	*ND*
lpg1924	lpp1899	lpl1888	lpc1378	lpa2783	lpw19631	PtVFX2014_11925	*ND*
lpg1933	lpp1914	lpl1903	lpc1406	lpa2811	lpw19721	PtVFX2014_13205	*lem15*
lpg1947	lpp1930	–	–	– $	lpw19951	–	*lem16*
lpg1948	–	–	–	–	–	–	*legLC4*
lpg1949	lpp1931	lpl1918	lpc1422	lpa2837	lpw19961	PtVFX2014_13280	*lem17*
lpg1950	lpp1932	lpl1919	lpc1423	lpa2838	lpw19971	PtVFX2014_13285	*ralF*
lpg1953	lpp1935	lpl1922	lpc1426	lpa2842	lpw20041	PtVFX2014_08220	*legC4*
lpg1958	lpp1940	–	–	–	–	–	*legL5*
lpg1959	lpp1941	# (lpl1932)	lpc1436	lpa2857	lpw20101	PtVFX2014_08135	*ND*
lpg1960	lpp1942	# (lpl1934)	lpc1437	lpa2859	lpw20111	PtVFX2014_08130	*lirA*
lpg1962	lpp1946	lpl1936	lpc1440	lpa2861	lpw20131	PtVFX2014_08120	*lirB*
lpg1963	–	–	Ψ ? (lpc1441/ lpc1442)	Ψ ? (lpa2862/ lpa2863)	–	–	*lirC/pieA*
lpg1964	–	–	–	–	–	–	*pieB/lirD*
lpg1965	–	–	– $	lpa2865	lpw20141	–	*lirE/pieC*
lpg1966	lpp1947	–	lpc1446	lpa2867	lpw20151	–	*pieD/lirF*
lpg1969	lpp1952	lpl1941	lpc1452	lpa2874	lpw20201	PtVFX2014_08095	*pieE*
lpg1972	lpp1955	lpl1950	lpc1459	lpa2884	lpw20291	PtVFX2014_08060	*pieF*
Ψ ? (lpg1975/ lpg1976)	lpp1959	lpl1953	lpc1462	lpa2889	lpw20351	PtVFX2014_08045	*ND*
lpg1978	lpp1961	lpl1955	lpc1464	lpa2892	lpw20371	PtVFX2014_08035	*setA*
lpg1986	lpp1967	lpl1961	lpc1469	lpa2898	lpw20431	PtVFX2014_07995	*ND*
lpg2050	lpp2033	lpl2028	lpc1536	lpa2992	lpw21141	Ψ ?	*ND*
lpg2131	–	–	–	–	–	# (PtVFX2014_14320)	*legA6*

lpg2137	lpp2076	lpl2066	lpc1586	lpa3060	lpw23101	PtVFX2014_14350	*legK2*
lpg2144	lpp2082	lpl2072	lpc1593	lpa3071	lpw23181	PtVFX2014_14385	*ankB/leg/AU13/ceg27*
lpg2147	lpp2086	lpl2075	lpc1596	lpa3076	lpw23211	PtVFX2014_14400	*mavC*
lpg2148	lpp2087	lpl2076	lpc1597	lpa3077	lpw23221	PtVFX2014_14405	*ND*
lpg2149	lpp2088	lpl2077	lpc1598	lpa3078	lpw23231	PtVFX2014_14410	*ND*
lpg2153	lpp2092	lpl2081	lpc1602	lpa3083	lpw23271	Ψ ? (PtVFX2014_14430/ PtVFX2014_14515)	*sdeC*
lpg2154	lpp2093	lpl2082	lpc1603	lpa3086	lpw23281	PtVFX2014_14510	*sdeC*
lpg2155	lpp2094	lpl2083	lpc1604	lpa3087	lpw23291	PtVFX2014_14505	*sidJ*
lpg2156	lpp2095	lpl2084	lpc1605	lpa3088	lpw23301	Ψ ? (PtVFX2014_01940)	*sdeB*
lpg2157	lpp2096	lpl2085	lpc1618	lpa3037	lpw23331	PtVFX2014_01930	*sdeC*
lpg2160	lpp2099	lpl2088	lpc1621	lpa3100	lpw23361	PtVFX2014_01915	*ND*
lpg2164	lpp2102	lpl2091	lpc1624	lpa3104	lpw23421	PtVFX2014_01900	*ND*
lpg2166	lpp2104	lpl2093	lpc1626	lpa3107	lpw23451	PtVFX2014_01890	*lem19*
lpg2176	lpp2128	lpl2102	lpc1635	lpa3118	lpw23561	PtVFX2014_01845	*legS2*
lpg2199	lpp2149	lpl2123	lpc1663	lpa3157	lpw23811	PtVFX2014_01735	*cegC4*
lpg2200	lpp2150	lpl2124	lpc1664	lpa3158	lpw23821	PtVFX2014_01730	*cegC4*
lpg2215	lpp2166	lpl2140	lpc1680	lpa3179	lpw24011	PtVFX2014_01655	*legA2*
lpg2216	lpp2167	lpl2141	lpc1681	lpa3180	lpw24021	PtVFX2014_01650	*lem20*
lpg2222	lpp2174	lpl2147	lpc1689	lpa3191	lpw24081	PtVFX2014_01620	*lpnE*
lpg2223	lpp2175	# (lpl2149)	lpc1691	lpa3196	lpw24091	PtVFX2014_01615	*ND*
lpg2224	–	–	–	–	–	–	*ppgA*
lpg2239	lpp2192	–	–	–	lpw24261	–	*ND*
lpg2244	lpp2198	lpl2170	lpc1713	lpa3231	lpw24331	PtVFX2014_01515	*cetLP5*
lpg2248	lpp2202	lpl2174	lpc1717	lpa3237	lpw24371	PtVFX2014_01495	*lem21*
lpg2271	lpp2225	lpl2197	lpc1740	lpa3268	lpw24611	PtVFX2014_01380	*ND*
lpg2283	lpp2237	lpl2209	lpc1752	lpa3282	lpw24741	PtVFX2014_01320	*cetLP6*
lpg2298	lpp2246	lpl2217	lpc1763	lpa3296	lpw24841	PtVFX2014_01265	*ylfA/legC7*
lpg2300	lpp2248	lpl2219	lpc1765	lpa3298	lpw24871	PtVFX2014_01255	*ankH/legA3/ankW*
lpg2311	lpp2259	lpl2230	lpc1776	lpa3312	lpw24981	PtVFX2014_01200	*ceg28*

lpg2322	lpp2270	lpl2242	lpc1789	lpa3328	lpw25121	PtVFX2014_01140	*ankK/legA5*
lpg2327	lpp2275	lpl2247	lpc1794	lpa3335	lpw25181	PtVFX2014_01115	*ND*
lpg2328	lpp2276	lpl2248	lpc1795	lpa3336	lpw25191	PtVFX2014_01110	*lem22*
lpg2344	lpp2292	lpl2265	lpc1812	lpa3355	lpw25371	PtVFX2014_01030	*mavE*
lpg2351	lpp2300	lpl2273	lpc1820	lpa3367	lpw25461	PtVFX2014_00990	*mavF*
lpg2359	lpp2308	lpl2281	lpc1828	lpa3376	lpw25561	PtVFX2014_00950	*ND*
lpg2370	–	–	–	–	–	–	*ND*
lpg2372	lpp3009	–	lpc3248	lpa4300	–	–	*ND*
lpg2375	lpp2440	–	lpc2117	lpa3432	–	PtVFX2014_00795	*pelF*
lpg2382	lpp2444	lpl2300	lpc2108	lpa3446	lpw25841	PtVFX2014_00740	*ND*
lpg2391	lpp2458	lpl2315	lpc2086	lpa3485	lpw26021	PtVFX2014_00685	*sdbC*
lpg2392	lpp2459	lpl2316	lpc2085	lpa3486	lpw26041	PtVFX2014_00680	*legL6*
lpg2400	–	lpl2323	–	–	lpw26121	–	*legL6*
lpg2406	lpp2472	lpl2329	lpc2070	lpa3506	lpw26191	PtVFX2014_00615	*lem23*
lpg2407	lpp2474	–	lpc2069	lpa3507	–	PtVFX2014_00610	*ND*
lpg2409	lpp2476	lpl2332	lpc2067	lpa3511	lpw26241	PtVFX2014_00600	*ceg29*
lpg2410	lpp2479	lpl2334	lpc2065	lpa3513	lpw26261	PtVFX2014_00590	*vpdA*
lpg2411	lpp2480	lpl2335	lpc2064	lpa3515	lpw26281	PtVFX2014_00585	*lem24*
–	lpp2486	–	–	–	–	PtVFX2014_00550	*ND*
lpg2416	# (lpp2484)	lpl2339	lpc2057	lpa3527	lpw26351	PtVFX2014_00560	*legA1*
lpg2420	–	lpl2343	lpc2056	lpa3529	lpw26391	–	*ND*
lpg2422	lpp2487	lpl2345	lpc2055	lpa3530	lpw26401	PtVFX2014_00545	*lem25*
lpg2424	lpp2489	lpl2347	lpc2053	lpa3532	lpw26421	PtVFX2014_00535	*mavG*
lpg2425	lpp2491	lpl2348	lpc2051	lpa3537	lpw26431	PtVFX2014_00525	*mavH*
lpg2433	lpp2500	lpl2353	lpc2043	lpa3548	lpw26521	PtVFX2014_00485	*ceg30*
lpg2434	lpp2501	lpl2355	lpc2042	lpa3550	lpw26531	PtVFX2014_00480	*ND*
lpg2443	lpp2510	lpl2363	lpc2033	lpa3562	lpw26631	PtVFX2014_00430	*ND*
lpg2444	lpp2511	lpl2364	lpc2032	lpa3563	lpw26641	PtVFX2014_00425	*mavI*
lpg2452	lpp2517	lpl2370	lpc2026	lpa3574	lpw26701	PtVFX2014_00395	*ankF/legA14/ceg31*
lpg2456	lpp2522	lpl2375	lpc2020	lpa3583	lpw26751	PtVFX2014_00370	*ankD/legA15*
lpg2461	lpp2527	lpl2380	lpc2015	lpa3589	lpw26801	PtVFX2014_00345	*ND*
lpg2464	–	lpl2384	–	–	lpw26851	–	*sidM/drrA*

lpg2465	–	lpl2385	–	–	lpw26861	–	*sidD*
lpg2482	lpp2546	lpl2402	lpc1996	lpa3615	lpw27041	PtVFX2014_00250	*sdbB*
lpg2490	lpp2555	lpl2411	lpc1987	lpa3628	lpw27131	PtVFX2014_00205	*lepB*
lpg2498	lpp2566	lpl2420	lpc1975	lpa3646	lpw27241	PtVFX2014_00060	*mavJ*
lpg2504	lpp2572	lpl2426	lpc1967	lpa3658	lpw27301	PtVFX2014_00090	*sidI/ceg32*
lpg2505	lpp2573	lpl2427	lpc1966	lpa3659	lpw27311	PtVFX2014_00095	*ND*
lpg2508	lpp2576	lpl2430	Ψ ? (lpc1962/ lpc1963)	Ψ ? (lpa3665/ lpa3666)	lpw27341	PtVFX2014_00110	*sdjA*
lpg2509	lpp2577	lpl2431	lpc1961	lpa3667	lpw27351	PtVFX2014_00115	*sdeD*
lpg2510	lpp2578	lpl2432	lpc1960	lpa3668	lpw27361	Ψ	*sdcA*
lpg2511	lpp2579	lpl2433	lpc1959	lpa3669	lpw27371	–	*sidC*
lpg2523	–	–	–	–	lpw27501	–	*lem26*
lpg2525	–	–	–	–	–	–	*mavK*
lpg2526	lpp2591	lpl2446	lpc1946	lpa3687	lpw27521	PtVFX2014_00185	*mavL*
lpg2527	lpp2592	lpl2447	lpc1944	lpa3688	lpw27531	PtVFX2014_00190	*ND*
lpg2529	lpp2594	lpl2449	lpc1942	lpa3692	lpw27551	PtVFX2014_08890	*lem27*
lpg2538	lpp2604	lpl2459	lpc1930	lpa3706	lpw27671	PtVFX2014_08820	*ND*
lpg2539	lpp2605	lpl2460	lpc1929	lpa3707	lpw27681	PtVFX2014_08815	*ND*
lpg2541	lpp2607	lpl2462	lpc1927	lpa3710	lpw27701	PtVFX2014_08810	*ND*
lpg2546	lpp2615	–	lpc1919	lpa3727	lpw27791	PtVFX2014_08795	*ND*
lpg2552	lpp2622	lpl2473	lpc1911	lpa3738	lpw27871	PtVFX2014_08760	*ND*
lpg2555	lpp2625	lpl2480	lpc1908	lpa3743	lpw27901	–	*ND*
lpg2556	lpp2626	lpl2481	lpc1906	lpa3745	lpw27911	–	*legK3*
lpg2577	lpp2629	lpl2499	lpc0570	lpa3768	lpw28241	PtVFX2014_06460	*mavM*
lpg2584	lpp2637	lpl2507	lpc0561	lpa3779	lpw28321	PtVFX2014_06500	*sidF*
lpg2588	lpp2641	lpl2511	lpc0557	lpa3784	lpw28361	PtVFX2014_06520	*legS1*
lpg2591	lpp2644	lpl2514	lpc0551	lpa3790	lpw28391	PtVFX2014_06535	*ceg33*
lpg2603	lpp2656	lpl2526	lpc0539	lpa3807	lpw28521	PtVFX2014_06595	*lem28*
lpg2628	lpp2681	lpl2553	lpc0513	lpa3846	lpw28781	PtVFX2014_06720	*ND*
lpg2637	lpp2690	lpl2562	lpc0503	lpa3859	lpw28871	PtVFX2014_06765	*ND*
lpg2638	lpp2691	lpl2563	lpc0502	lpa3861	lpw28891	PtVFX2014_06770	*mavV*
lpg2692	lpp2746	lpl2619	lpc0444	lpa3929	lpw29461	PtVFX2014_07050	*ND*

lpg2694	lpp2748	lpl2621	lpc0442	lpa3931	lpw29481	PtVFX2014_07060	*legD1*
lpg2718	lpp2775	lpl2646	lpc0415	lpa3966	lpw29771	PtVFX2014_07195	*wipA*
lpg2720	lpp2777	lpl2648	lpc0413	lpa3968	lpw29791	PtVFX2014_07205	*legN*
lpg2744	lpp2800	lpl2669	lpc0386	lpa4004	lpw30031	–	*ND*
lpg2745	lpp2801	lpl2670	lpc0385	lpa4005	lpw30041	PtVFX2014_07320	*ND*
lpg2793	lpp2839	lpl2708	lpc3079	lpa4063	lpw30471	PtVFX2014_14785	*lepA*
lpg2804	lpp2850	lpl2719	lpc3090	lpa4076	lpw30591	PtVFX2014_14835	*lem29*
lpg2806	lpp2852	lpl2721	lpc3092	lpa4078	lpw30611	PtVFX2014_14845	*cetLP7*
lpg2815	lpp2867	lpl2730	lpc3101	lpa4089	lpw30711	PtVFX2014_14890	*mavN*
lpg2826	–	lpl2741	lpc3113	lpa4104	lpw30831	PtVFX2014_14945	*ceg34*
lpg2828	lpp2882	lpl2743	lpc3115	lpa4109	lpw30851	PtVFX2014_14955	*ND*
lpg2829	– $	–	–	–	lpw30861	–	*sidH*
lpg2830	lpp2887	–	–	–	lpw30881	–	*lubX/legU2*
lpg2831	lpp2888	–	–	–	lpw30891	–	*VipD*
lpg2832	lpp2889	lpl2744	lpc3116	lpa4110	lpw30921	PtVFX2014_14960	*ND*
lpg2844	lpp2903	lpl2756	lpc3128	lpa4133	–	PtVFX2014_15020	*ND*
lpg2862	–	–	–	–	–	–	*Lgt2/legC8*
lpg2874	lpp2933	lpl2787	lpc3160	lpa4176	lpw31411	PtVFX2014_15175	*ND*
lpg2879	lpp2938	lpl2792	lpc3165	lpa4186	lpw31471	PtVFX2014_15200	*ND*
lpg2884	lpp2943	lpl2797	lpc3170	lpa4193	lpw31531	PtVFX2014_15225	*ND*
lpg2885	lpp2944	lpl2798	lpc3171	lpa4194	lpw31541	PtVFX2014_15230	*ND*
lpg2888	lpp2947	lpl2801	lpc3174	lpa4199	lpw31571	PtVFX2014_15245	*ND*
lpg2907	lpp2976	lpl2824	lpc3210	lpa4248	lpw31821	PtVFX2014_15365	*mavW*
lpg2912	lpp2980	lpl2830	lpc3214	lpa4255	lpw31931	–	*ND*
lpg2936	lpp3004	lpl2865	lpc3243	lpa4293	lpw32251	PtVFX2014_15510	*ND*
lpg2975	lpp3047	lpl2904	lpc3290	lpa4358	lpw32621	PtVFX2014_10160	*ND*
lpg2999	lpp3071	lpl2927	lpc3315	lpa4395	lpw32851	PtVFX2014_10040	*legP*
lpg3000	lpp3072	lpl2928	lpc3316	lpa4397	lpw32861	PtVFX2014_10035	*ND*

[a] Analysis of presence/absence of 303 known Dot/Icm substrates coding genes that were recently gathered in the literature[21-22].

[b] Loci designations are based on the genome annotation of the respective strains (accession numbers are in Supplemental Table 1).

Ψ Pseudogene, i.e., sequence is present and the gene is annotated as pseudogene. This kind of genes was counted in the overall repertoire displayed in Figure 5, since its putative inactivaction does not mean a "true" absence (i.e., lack of homolog sequence).

Ψ ? Putative pseudogene, i.e., sequence is present, but : i) annotation is missing; ii) it is not finished or not manually curated (i.e., it is splitted in two contigs or by undefined nucleotides); or iii) it is annotated in two contiguous genes that overlap the entire predicted effector coding gene. This kind of genes was counted in the overall repertoire displayed in Figure 5, since its mis-annotation/inactivation does not mean a "true" absence (i.e., lack of homolog sequence).
– or # Absent or the region displaying homology is clearly shorter than the predicted gene (although a small ORF may be annotated as pseudogene). This kind of genes was not counted in the overall repertoire displayed in Figure 5.
– $; Due to an indel event, the predicted effector coding gene was splitted in more than one small protein-coding gene. This kind of genes was not counted in the overall repertoire displayed in Figure 5.
ND, not defined.

References of the Supplementary Information

1. Chien, M. *et al.* The genomic sequence of the accidental pathogen Legionella pneumophila. *Science* **305**, 1966-1968 (2004).
2. Underwood, A. P., Jones, G., Mentasti, M., Fry, N. K. & Harrison, T. G. Comparison of the Legionella pneumophila population structure as determined by sequence-based typing and whole genome sequencing. *BMC Microbiol* **13**, 302 (2013).
3. Cazalet, C. *et al.* Evidence in the Legionella pneumophila genome for exploitation of host cell functions and high genome plasticity. *Nat Genet* **36**, 1165-1173 (2004).
4. Khan, M. A. *et al.* Comparative Genomics Reveal That Host-Innate Immune Responses Influence the Clinical Prevalence of Serogroups. *PLoS One* **8**, e67298 (2013).
5. Glockner, G. *et al.* Identification and characterization of a new conjugation/type IVA secretion system (trb/tra) of Legionella pneumophila Corby localized on two mobile genomic islands. *Int J Med Microbiol* **298**, 411-428 (2008).
6. Ginevra, C. *et al.* Lorraine strain of Legionella pneumophila serogroup 1, France. *Emerg Infect Dis* **14**, 673-675 (2008).
7. Edelstein, P. H. & Metlay, J. P. Legionella pneumophila goes clonal--Paris and Lorraine strain-specific risk factors. *Clin Infect Dis* **49**, 192-194 (2009).
8. D'Auria, G., Jimenez-Hernandez, N., Peris-Bondia, F., Moya, A. & Latorre, A. Legionella pneumophila pangenome reveals strain-specific virulence factors. *BMC Genomics* **11**, 181 (2010).
9. Schroeder, G. N. *et al.* Legionella pneumophila strain 130b possesses a unique combination of type IV secretion systems and novel Dot/Icm secretion system effector proteins. *J Bacteriol* **192**, 6001-6016 (2010).
10. Gomez-Valero, L. *et al.* Extensive recombination events and horizontal gene transfer shaped the Legionella pneumophila genomes. *BMC Genomics* **12**, 536 (2011).
11. Ko, K. S. *et al.* Population genetic structure of Legionella pneumophila inferred from RNA polymerase gene (rpoB) and DotA gene (dotA) sequences. *J Bacteriol* **184**, 2123-2130 (2002).
12. Amaro, F., Gilbert, J. A., Owens, S., Trimble, W. & Shuman, H. A. Whole-genome sequence of the human pathogen Legionella pneumophila serogroup 12 strain 570-CO-H. *J Bacteriol* **194**, 1613-1614 (2012).
13. Ma, J., He, Y., Hu, B. & Luo, Z. Q. Genome Sequence of an Environmental Isolate of the Bacterial Pathogen Legionella pneumophila. *Genome Announc* **1** (2013).
14. Brenner, D. J. *et al.* Legionella pneumophila serogroup Lansing 3 isolated from a patient with fatal pneumonia, and descriptions of L. pneumophila subsp. pneumophila subsp. nov., L. pneumophila subsp. fraseri subsp. nov., and L. pneumophila subsp. pascullei subsp. nov. *J Clin Microbiol* **26**, 1695-1703 (1988).
15. Brzuszkiewicz, E. *et al.* Legionella oakridgensis ATCC 33761 genome sequence and phenotypic characterization reveals its replication capacity in amoebae. *Int J Med Microbiol* **303**, 514-528 (2013).
16. Gaia, V. *et al.* Consensus sequence-based scheme for epidemiological typing of clinical and environmental isolates of Legionella pneumophila. *J Clin Microbiol* **43**, 2047-2052 (2005).
17. Gaia, V., Fry, N. K., Harrison, T. G. & Peduzzi, R. Sequence-based typing of Legionella pneumophila serogroup 1 offers the potential for true portability in legionellosis outbreak investigation. *J Clin Microbiol* **41**, 2932-2939 (2003).
18. Ratzow, S., Gaia, V., Helbig, J. H., Fry, N. K. & Luck, P. C. Addition of neuA, the gene encoding N-acylneuraminate cytidylyl transferase, increases the discriminatory ability of the consensus sequence-based scheme for typing Legionella pneumophila serogroup 1 strains. *J Clin Microbiol* **45**, 1965-1968 (2007).
19. Cao, B., Yao, F., Liu, X., Feng, L. & Wang, L. Development of a DNA microarray method for detection and identification of all 15 distinct O-antigen forms of Legionella pneumophila. *Appl Environ Microbiol* **79**, 6647-6654 (2013).
20. Cazalet, C. *et al.* Multigenome analysis identifies a worldwide distributed epidemic Legionella pneumophila clone that emerged within a highly diverse species. *Genome Res* **18**, 431-441 (2008).

21. Gomez-Valero, L., Rusniok, C., Cazalet, C. & Buchrieser, C. Comparative and functional genomics of legionella identified eukaryotic like proteins as key players in host-pathogen interactions. *Front Microbiol* **2**, 208 (2011).
22. Gomez-Valero, L. *et al.* Comparative analyses of Legionella species identifies genetic features of strains causing Legionnaires' disease. *Genome Biol* **15**, 505 (2014).
